# Integrating Targeted and Untargeted Metabolomics to Investigate the Processing Chemistry of Polygoni Multiflori Radix

**DOI:** 10.3389/fphar.2018.00934

**Published:** 2018-08-28

**Authors:** Li Liang, Jun Xu, Wen-Wen Zhou, Eric Brand, Hu-Biao Chen, Zhong-Zhen Zhao

**Affiliations:** School of Chinese Medicine, Hong Kong Baptist University, Kowloon, Hong Kong

**Keywords:** polygoni multiflori radix, targeted and untargeted metabolomics, processing chemistry, UPLC-QTOF-MS/MS, UPLC-QqQ-MS/MS

## Abstract

Polygoni Multiflori Radix (PMR, *Heshouwu* in Chinese), derived from the tuberous roots of *Polygonum multiflorum* Thunb., is a widely-used Chinese medicinal material. For traditional clinical use, raw PMR (RPMR) is processed by nine cycles of steaming and drying to generate processed PMR (PPMR); RPMR and PPMR have distinct medicinal purposes based on the theory of traditional Chinese medicine. While PMR has been processed for hundreds of years, including the present, the chemistry of that processing has not been well studied. In this study, targeted and untargeted metabolomics analyses using ultra-performance liquid chromatography-quadrupole/time-of-flight mass spectrometry (UPLC-QTOF-MS/MS) and ultra-performance liquid chromatography-quadrupole/triple quadrupole mass spectrometry (UPLC-QqQ-MS/MS) were integrated to investigate the processing chemistry of PMR. The results demonstrate that processing by nine cycles of steaming and drying qualitatively and quantitatively alters the chemical profile of PMR. Several mechanisms, namely hydrolysis, dehydration, isomerization, and Maillard reaction appear to be involved in the chemical transformation that occurs. The qualitative and quantitative data further suggest that nine cycles might be necessary for the preparation of PPMR, as PPMR that has been processed nine times shows significant differences in its chemical profile.

## Introduction

Polygoni Multiflori Radix (PMR, *heshouwu* in Chinese), derived from the tuberous roots of *Polygonum multiflorum* Thunb., is a widely-used Chinese medicinal material (CMM) (Chinese Pharmacopoeia Commission, 2015). For traditional clinical use, raw PMR (RPMR) is processed by nice cycles of steaming and sun-drying to generate processed PMR (PPMR) (Li, 2008). According to the theory of Chinese medicine, RPMR and PPMR are prescribed for distinct medicinal purposes (Han et al., 2009; Chinese Pharmacopoeia Commission, 2015). RPMR is used to resolve toxin and free the stool, while PPMR is regarded as a traditional tonic for its rejuvenation effects, with the function of supplying the liver and kidney, as well as strengthening the sinews and bones (Chinese Pharmacopoeia Commission, 2015). Modern pharmacological research has revealed that RPMR and PPMR have different bioactivities (Ye, 1987; Zhao et al., 2008). Nevertheless, the scientific basis involved in the processing of PMR is not yet well understood. The alternate steaming and sun-drying appears to transform certain bioactive components, changing the herb's therapeutic properties. While those transformations are taken as theoretically true, in modern pharmaceutical processing, these steps are being abbreviated such that most PPMR in the market today has been processed through only one cycle (Chinese Pharmacopoeia Commission, 2015). The resulting product is being used, but no one has evaluated it in terms of its chemistry to determine if it resembles PMR processed through the prescribed nine cycles. Research on processing chemistry to elucidate how processing alters the chemical components is the first step to establishing a scientific basis for PMR processing.

It has been well demonstrated that several types of secondary metabolites are the major chemical components of PMR; of these, stilbene glucosides, anthraquinones and polyphenols, are most representative (Yao et al., 2006; Han et al., 2013; Lin et al., 2015b). By quantifying these secondary metabolites using liquid chromatography coupled with diode-array detectors or mass spectrometry (LC-DAD/MS), some previous studies investigated the processing chemistry of PMR. The results showed that processing indeed changed the contents of certain chemicals in PMR (Liang et al., 2010; Liu et al., 2011; Chen et al., 2016; Wang et al., 2016). For example, we previously found that the contents of two anthraquinones (emodin and physicon) in PMR were increased by the processing, while one stilbene glucoside named 2,3,5,4′-tetrahydroxystilbene-2-*O*-β-D-glucoside (THSG) was decreased (Liang et al., 2010). However, these studies are deficient in the following two aspects. Firstly, they selected several typical chemical components for the determination, and did not characterize the entire chemical profile of PMR. Secondly, and more importantly, these studies analyzed PPMR that had been processed by only one cycle of steaming and drying (Liang et al., 2010; Liu et al., 2011; Chen et al., 2016; Wang et al., 2016), which is different from the traditional processing technology of PMR which stipulates nine cycles of steaming and sun-drying (Li, 2008). Given these facts, further research is needed to fully elucidate the processing chemistry of PMR.

In recent years, metabolomics, both targeted and untargeted, has been widely applied to comprehensively investigate chemical variation in CMMs including processing. Untargeted metabolomics aims to qualitatively determine all measurable analytes in a sample, including chemical unknowns, while targeted metabolomics further absolutely quantifies a group of defined chemicals. The integration of targeted and untargeted metabolomics can offer deeper insights into the processing chemistry of CMMs by providing comprehensive qualitative and quantitative information of total secondary metabolites. Actually, metabolomics has been preliminarily attempted for investigating the processing chemistry of PMR (Yu et al., 2017). However, absolute quantification was not involved in this study. Moreover, the study only observed impacts of processing time, rather than processing cycles, on PMR chemical profiles. Thus, here we integrated targeted and untargeted metabolomics to further investigate the processing chemistry of PMR. The study was designed in the following steps. Step 1, PPMR was prepared from RPMR by nine cycles of steaming and sun-drying. Step 2, the secondary metabolite profiles of RPMR and PPMR were characterized and compared by untargeted metabolomics using ultra-performance liquid chromatography-quadrupole/time-of-flight mass spectrometry (UPLC-QTOF-MS/MS), in which the chemicals that were most significantly altered by the processing were statistically explored by multivariate statistical analysis. Step 3, 12 typical chemical components were quantitatively determined by targeted metabolomics using ultra-high-performance liquid chromatograph with triple quadrupole mass spectrometry (UHPLC-QqQ-MS/MS) to further observe their content variations in each processing cycle. Steps 4, based on the qualitative and quantitative results, the potential mechanisms involved in the processing-induced chemical transformations were proposed.

## Materials and methods

### Plant materials, chemicals, and reagents

RPMR materials were collected from Kaili City (Guizhou, China), the geo-authentic producing area of PMR, and were authenticated as the tuberous roots of *Polygonum multiflorum* Thunb. by Prof. Zhongzhen Zhao. The voucher specimens were deposited in the Chinese Medicines Centre, Hong Kong Baptist University.

Chemical standards including gallic acid, proanthocyanidin B1, proanthocyanidin B2, *trans*-2,3,5,4′-tetrahydroxylstilbene-2-*O*-β-D-glucoside (*trans*-THSG), emodin, physcion were ordered from Chengdu Must Bio-Technology Co., Ltd. (Chengdu, China); epicatechin, epicatechin-gallate, emodin-8-*O*-β-D-glucoside, physcion-8-*O*-β-D-glucoside were supplied by Chengdu Xunchen Biological Technology Co., Ltd. (Chengdu, China). Acetonitrile (HPLC grade) and methanol (HPLC grade) were supplied by E. Merck (Darmstadt, Germany); ammounium acetate (Sigma-aldrich, USA) was purchased; formic acid (HPLC grade) was purchased from Tedia (USA); ultra-pure water was prepared by a Mili-Q water purification system (Millipore, MA, USA).

### Sample preparation

#### Processing

RPMR (20 kg) was randomly divided into 8 groups. One tenth of each group were reserved as materials of RPMR; the rest was subsequently processed. To fix the specific factors involved in the processing, reports in the literature (Li, 2008; Liang et al., 2010; Liu et al., 2011; Chen et al., 2016; Wang et al., 2016) and the Chinese pharmacopeia (2015 edition) (Chinese Pharmacopoeia Commission, 2015) were referenced. Finally, 4 h steaming with black bean (put one layer of water-soaked black beans and one layer of RPMR slices in a pot, repeat the layers, and then steam over boiling water) and then 24 h sun-drying, repeated for a total of 9 cycles, were used as processing conditions. Samples were retained after each cycle, thus generating nine PPMR samples (PPMR1-PPMR9). The color of PPMR samples became darker after each processing cycle (Figure S1).

#### Extraction

For the extraction, our previous study was referenced (Liang et al., 2018). Each RPMR and PPMR sample was powdered, accurately weighed (0.1 g), and put into a 50-mL centrifugal tube with 20 mL 70% methanol. The mixture was then ultra-sonicated for 45 min at 60°C. After that, the solution was centrifuged at 3,000 rpm for 10 min to obtain the supernatant. The extraction procedure was repeated one more time. Then 5 mL 70% methanol was used to wash the residue. The three resultant solutions were combined and made up to 50 mL, which was then filtered through a 0.22 μm filter for further analysis.

### Untargeted metabolomics analysis

#### UHPLC-QTOF-MS/MS conditions

Untargeted metabolomics analysis was performed on an Agilent 6540 UHPLC-QTOF-MS/MS system (Agilent Technologies, USA). The chromatographic separation was achieved on a UHPLC C18 analytical column (2.1 × 100 mm, I.D. 1.7 μm, BEH) coupled with a C18 pre-column (2.1 × 5 mm, I.D.1.7 μm, ACQUITY UPLC BEH, Waters, USA). The elution was conducted under the following conditions: the mobile phase consisted of (A) water containing 0.1% formic acid and (B) acetonitrile containing 0.1% formic acid, and the gradient program was: 2–5% B (0–2 min); 5–70% B (2–16 min); 70–100% B (16–23 min); 100% B (23–26 min), with 4 min of balance back to 2% B. The injection volume was 3 μL, and the flow rate was 0.4 mL/min. The mass spectra were acquired in both negative and positive modes, *m/z* ranging from 100 to 1,700, ESI ion source. The dry gas (N_2_) flow rate was 8 L/min with the temperature at 300°C. The capillary voltage, nozzle voltage, and fragment voltage were set at 4500 V, 500 V and 150 V, respectively; while nebulizer pressure was 45 psi, and column temperature was set at 40°C.

#### Establishment of in-house database and peak characterization

Previously reported chemical components derived from RPMR and PPMR were collected and summarized in a *Microsoft Office Excel* table, and the table was applied to establish a compound database using Agilent Mass Hunter PCDL Manager software (Agilent Technologies, B.04.00, 2011). The database included compound name, chemical structure, molecular formula, weight and related references. Agilent Mass Hunter Work station software-Qualitative Analysis (version B.06.00, Agilent Technologies 2012) was used. Base Peak Chromatogram (BPC with *m/z* ranging from 100 to 1,000) was selected to show the results. The empirical molecular formulas were deduced by a comparison of the accurately measured mass values and the theoretical exact mass values of protonated and deprotonated molecular ions and/or fragment ions with a mass accuracy <10 ppm, and then matched with known compounds in the database using the “*Find*” function. For those compounds which were not listed in the PCDL database, possible formulas were deduced according to the molecular mass, fragment ions and mass accuracy.

#### Multivariate statistical analysis

The raw data of UPLC-QTOF-MS/MS were processed by DA Reprocessor (version B.06.00, Agilent Technologies, Inc. 2012), and the parameters were set as follows: retention time range from 0 to 30 min, mass range 100–1,000 Da, minimum absolute height 5,000 counts, mass tolerance 10 ppm, peak spacing tolerance within 0.0025 *m/z* and the limit assigned charge states to a maximum of 5. The generated data was then processed for principal component analysis (PCA) and Volcano Plot analysis by Mass Profiler Professional (2.0 vision, Agilent Technologies, Inc. 2009). The Volcano Plot was used to provide information regarding the differential abundance between raw and nine cycles processing samples based on *p*-value (<0.05) and fold-change at 3.

### Targeted metabolomics analysis

#### UHPLC-QqQ-MS/MS conditions

The targeted metabolomics analysis was conducted on an Agilent 6460 UHPLC-QqQ-MS/MS (Agilent Technologies, USA) with ESI ion source. The UHPLC-QqQ-MS/MS conditions were optimized based on our previous study (Liang et al., 2018). The chromatographic separations was achieved on a UHPLC C18 analytical column (2.1 × 100 mm, I.D. 1.7 μm, BEH) coupled with a C18 pre-column (2.1 × 5 mm, I.D.1.7 μm, ACQUITY UPLC BEH, Waters, USA) by two different conditions: (1) the mobile phase consisted of (A) water containing 0.1% formic acid and (B) acetonitrile containing 0.1%, and the gradient program was: 2% B (0–0.5 min); 5–15% B, (0.5–2 min); 15–40% B (2–8 min); 40–100% B (8–12 min); 100% B, (12–15 min), with 3 min of balance back to 2% B. The flow rate was 0.35 mL/min, and the injection volume was 2 μL. The mass spectra were acquired in negative mode, and the parameters were as follows: dry gas (N_2_) flowrate 8 L/min with the temperature at 350°C; sheath gas flow 8 L/min with heater at 350°C; nebulizer pressure, 45 psi; capillary voltage 3,500 V for ESI, 500 V charging, and with a dwell time of 20 ms for each ion pair. Other details are shown in **Table 2**; (2) the mobile phase consisted of 3 mM ammonium acetate in water (A) and methanol (B), and the gradient program was: 0–7 min, 35–100% B; 7–9 min, 100% B), with 3 min of balance back to 35% B. The flow rate was 0.35 mL/min; the injection volume was 2 μL; the column temperature was 60°C. Negative mode was selected for the mass spectra, and the details of parameters were set as: dry gas (N_2_) flowrate 7 L/min with temperature at 300°C; sheath gas flow 8 L/min with the sheath gas heater 350°C; nebulizer pressure, 45 psi; 500 V charging, capillary voltage 3,500 V for ESI, and with a dwell time of 40 ms for each ion pair. Other details are shown in **Table 2**.

#### Quantitative method validation

The UPLC-QqQ-MS/MS methods for quantitative determination of the 12 chemical compounds were validated with regard to linearity, sensitivity, precision, repeatability, stability and recovery.

The stock solutions of reference compounds were diluted with methanol to yield a series of appropriate concentration solutions for the construction of the calibration curves. The limits of detection (LODs) and limits of quantification (LOQs) were determined with signal-to-noise (*S/N*) ratios of 3 and 10, respectively.

The intra-day and inter-day variations were selected to determine the precision of the assay method. To assess intra-day variation, PPMR1 sample was extracted and analyzed for six replicates within 1 day; and to assess inter-day variation, the same sample was analyzed in triplicates in for two successive days. For repeatability, PPMR1 was extracted six times; the six extracts were analyzed, and the variation was used for repeatability evaluation. Stability test was performed by analyzing the extract of PPMR1 at 0, 2, 4, 8, 12, 24, and 72 h, respectively.

As for the recovery validation, 0.1 g of each of PPMR1 with known contents of the target analyses were accurately weighed. Then each was spiked with different amounts (low, middle and high level) of reference standards (gallic acid: 56.00, 70.00, and 84.00 μg; proanthocyanidin B1: 16.00, 21.00, and 25.00 μg; proanthocyanidin B2: 4.00, 5.00, and 6.00 μg; trans-THSG: 3.30, 4.10, and 4.90 mg; epicatechin: 29.00, 36.00, and 43.00 μg; epicatechin-3-gallate: 19.00, 24.00, and 28.00 μg; emodin-8-*O*-β-D-glucoside: 132.00, 166.00, and 199.00 μg; physcion-8-*O*-β-D-glucoside: 75.00, 93.00, and 112.00 μg; emodin: 29.00, 36.00, and 43.00 μg; physcion: 12.00, 15.00, and 18.00 μg, in solutions), then extracted according to the sample preparation procedure listed in section Extraction and analyzed in triplicate.

#### Quantitative data analysis

Three samples were taken from each processing cycle in order to assess the variation at each processing cycle; each sample was then analyzed three times, and the results of all nine analyses were averaged. Data were processed by Agilent Mass Hunter Work station software-Quantitative Analysis (version B.06.00, Build 6.0.388.1, Agilent Technologies, Inc. 2008). The charts of results were produced by GraphPad Prism 5 software (GraphPad, USA).

## Results and discussion

### Untargeted metabolomics analysis

#### Optimization of UHPLC-QTOF- MS/MS analytical conditions

LC-QTOF-MS/MS is one of the most frequently used techniques for untargeted metabolomics analysis of CMMs. In a QTOF-MS/MS analysis, each precursor ion selected by the quadrupole mass analyzer is dissociated in the collision cell, and the generated fragment ions are then successively detected by the TOF analyzer. The principle of QTOF-MS/MS allows it to effectively detect a wide range of chemicals in a few minutes. Furthermore, it can provide accurate mass measurement and sufficient fragment information for secondary metabolite identification (Chernushevich et al., 2001). In this study, untargeted metabolomics analysis was therefore performed by UHPLC-QTOF-MS/MS. To achieve more fragment information for chemical components identification, both negative and positive ion modes were used. For chromatographic separation, different mobile phases were compared, including two organic phases: acetonitrile and methanol, as well as two additives: formic acid and 3 mM ammonium acetate. Finally, 0.1% formic acid–water and 0.1% formic acid–acetonitrile was selected because they can elute more chemical compounds with better separation, as well as it generated higher value of the total ion abundances (Figure S2).

#### Compounds identification

**Figure 1:**
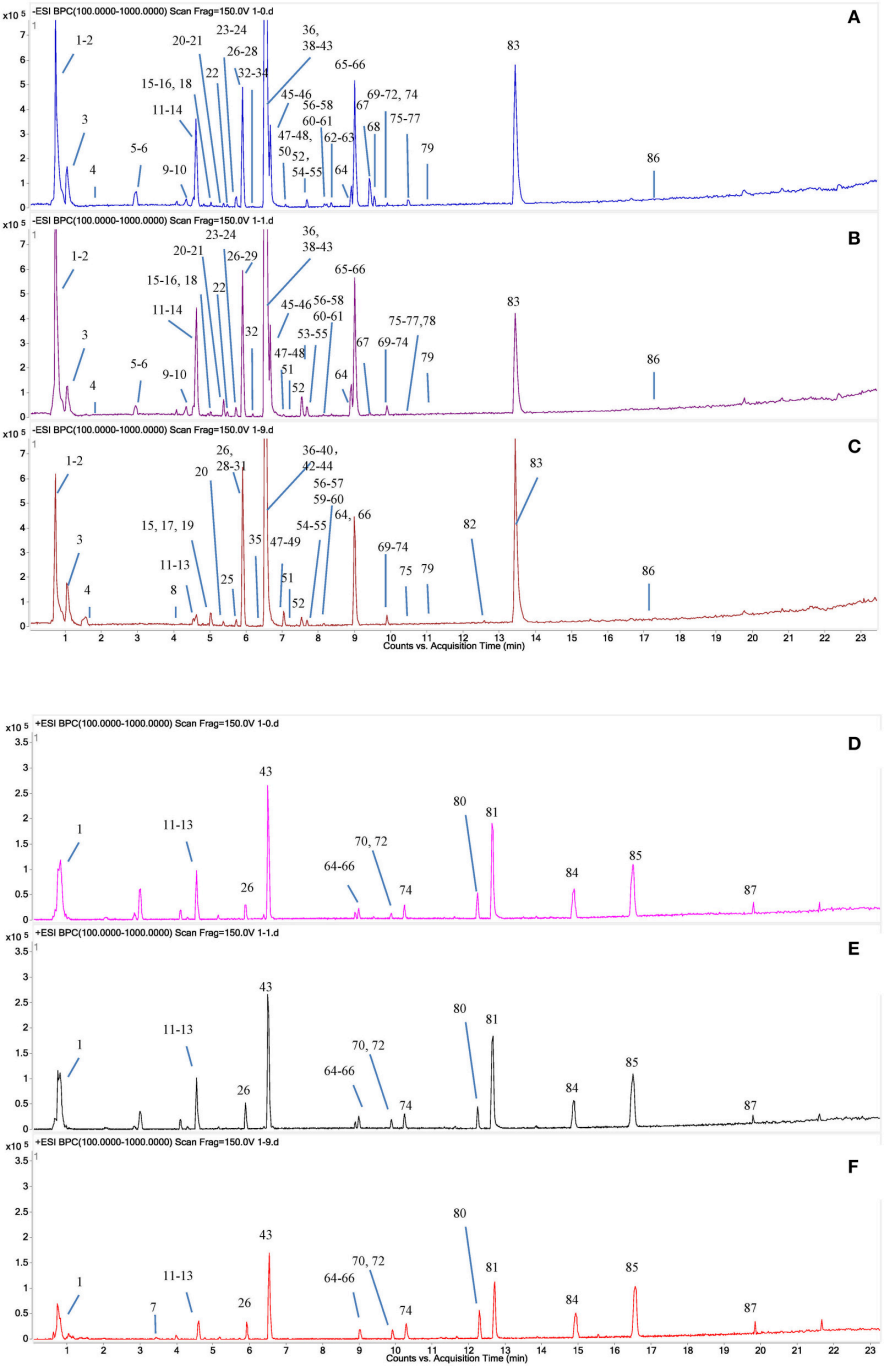
Typical UPLC-QTOF-MS/MS chromatograms of secondary metabolites in PMR. **(A,D)** RPMR; **(B,E)** PPMR1; **(C,F)** PPMR9; **(A–C)** negative mode; **(D–F)** positive mode. The peak numbers represent the same meanings as in Table 1.

A total of 87 compounds were definitely or tentatively identified in RPMR and PPMR by chemical standards and/or the established in-house database (Table 1; Qiu et al., 2013; Lin et al., 2015a; Wang et al., 2017). The representative LC-MS base peak chromatograms (BPCs) for RPMR and PPMR are shown in Figure 1. Three types of chemicals, namely stilbenes, anthraquinones, and catechin, were identified as the major secondary metabolites of RPMR and PPMR, and typical compounds of these three types of chemicals are used as examples here to explain the mass fragmentation pathways.

**Table 1:** Identification of the major secondary metabolites in RPMR and PPMR samples by UHPLC-QTOF-MS/MS.

**Peak No**.	**Rt (min)**	**Identity**	**Molecular formula**	**Molecular ions/adduct ions**	***m/z***			**Fragment ions of [M+H]^+^/[M–H]^−^(mass accuracy, ppm)**	**Occurrence in PMR (processing cycles)**
					**Mean measured mass (Da)**	**Theoretical exact mass (Da)**	**Mass accuracy (ppm)**		
1	0.76	Cephulac	C_12_H_22_O_11_	[M–H] ^−^ [M + Na] ^+^	341.1092 365.1055	341.1089 365.1054	0.9 0.3	179.0565 [M-H-Glu]^−^ (5.0) 119.0349 [ M-H-Glu-CH_2_COOH]^−^ (4.2)	0–9
2	0.76	Hydroxysuccinic acid	C_4_H_6_O_5_	[M–H]^−^	133.0140	133.0142	−1.5	115.0033 [M-H-H_2_O]^−^ (−3.4)	0–9
3	1.05	Hydocerol A	C_6_H_8_O_7_	[M–H]^−^	191.0195	191.0197	−1.0	111.0086 [M-H-CO_2_-2H_2_O]^−^ (3.6)	0–9
4	1.49	Gallic acid	C_7_H_6_O_5_	[M–H]^−^	169.0138	169.0142	−2.4	125.0236 [M-H-CO_2_]^−^ (−2.4) 107.0141 [M-H-CO_2_-H_2_O]^−^ (7.5)	0–9
5	2.91	Gallocatechin	C_15_H_14_O_7_	[M–H] ^−^	305.0666	305.0667	−0.3	179.0357 [M-H-C_6_H_6_O_3_]^−^ (1.8) 125.0242 [M-H-C_9_H_8_O_4_]^−^ (2.4)	0–4
6	3.00	Unknown	C_11_H_18_N_2_O_5_	[M + H]^−^	259.1285	259.1288	−1.2	130.0497 [M-H-C_6_H_11_NO_2_]^−^ (−1.5)	0–7
7	3.41	5-HMF	C_6_H_6_O_3_	[M + H] ^+^	127.0384	127.0390	4.7		7–9
8	3.98	3-Hydroxybenzoic acid	C_7_H_6_O_3_	[M–H] ^−^	137.0246	137.0244	1.5	108.0201 [M-H-CHO]^−^ (−2.4)	2–9
9	4.34	Proanhocyanidin B1	C_30_H_26_O_12_	[M–H]^−^	577.1353	577.1351	0.3	425.0863 [M-H-C_8_H_8_O_3_]^−^ (−2.4) 407.0773 [M-H-C_8_H_8_O_3_-H_2_O]^−^ (1.5) 289.0726 [M-H-C_15_H_14_O_6_]^−^ (4.8)	0–4
10	4.35	Epigallocatechin	C_15_H_14_O_7_	[M–H]^−^	305.0670	305.0667	1.0	179.0348 [M-H-C_6_H_6_O_3_]^−^ (1.1) 125.0242 [M-H-C_9_H_8_O_4_]^−^ (−1.6)	1–4
11	4.54	2-Vinyl-1H-indole-carboxylic acid	C_11_H_9_NO_2_	[M–H]^−^ [M + H] ^+^	186.0558 188.0706	186.0561 188.0706	−1.6 0	142.0662 [M-H-CO_2_]^−^ (3.5) 116.0615 [M-H-CO_2_-CN]^−^ (−9.5)	0–9
12	4.54	Hypaphorine	C_14_H_18_N_2_O_2_	[M–H] ^−^ [M + H] ^+^	245.1294 247.1434	245.1296 247.1441	−0.8 −2.8	142.0667 [M-H-N(CH_3_)_3_-CO_2_]^−^ (−3.5)	0–9
13	4.61	Catechin	C_15_H_14_O_6_	[M–H] ^−^ [M + H] ^+^	289.0720 291.0861	289.0718 291.0863	0.7 −1.7	245.0803 [M-H-C_2_H_3_OH]^−^ (−4.4) 151.0396 [M-H-C_7_H_6_O_3_]^−^ (0.7) 205.0489 [M-H-C_2_H_3_OH-C_2_OH]^−^ (1.8)	0–9
14	4.81	Bilobalide	C_15_H_18_O_8_	[M–H]^−^	325.0927	325.0929	−0.6	289.0714 [M-H-2H_2_O] ^−^ (−0.7) 245.0815 [M-H-2H_2_O- CO_2_]^−^ (1.4)	0–1
15	5.01	Salicylaldehyde	C_7_H_6_O_2_	[M-H]–	121.0291	121.0295	−3.3		0–9
16	5.07	Proanhocyanidin B2	C_30_H_26_O_12_	[M-H] ^−^	577.1349	577.1351	−0.3	425.0871 [M-H-C_8_H_8_O_3_]^−^ (−0.5) 407.0776 [M-H-C_8_H_8_O_3_-H_2_O]^−^ (2.2) 289.0735 [M-H-C_15_H_14_O_6_]^−^ (8.0)	0–2
17	5.11	Liquiritigenin-hexose-xyl/ara	C_26_H_30_O_13_	[M–H] ^−^	549.1602	549.1614	−2.2	459.1259 [M-H-C_3_H_6_O_3_]^−^ (8.3) 387.1098 [M-H-Glu]^−^ (−3.4)	4–9
18	5.20	Polygonumoside E	C_19_H_22_O_9_	[M–H] ^−^	393.1186	393.1191	−1.3	231.0653 [M-H-Glu] ^−^ (−0.5) 189.0556 [M-H-Glu-C_3_H_6_] ^−^ (−0.5)	0–2
19	5.20	isomer Polygonimitin C	C_26_H_32_O_14_	[M–H] ^−^	567.1713	567.1719	−1.1	549.1589 [M-H-H_2_O] ^−^ (−1.3)	4–9
20	5.37	Epicatechin	C_15_H_14_O_6_	[M–H] ^−^	289.0708	289.0718	−3.5	245.0828 [M-H-C_2_H_3_OH]^−^ (5.7) 205.0495 [M-H-C_2_H_3_OH-C_2_OH]^−^ (3.0) 151.0401 [M-H-C_7_H_6_O_3_]^−^ (1.8)	0–9
21	5.47	Procyanidin B1-3-*O*-gallate	C_37_H_30_O_16_	[M–H] ^−^	729.1460	729.1461	−0.1	577.1355 [M-H-C_8_H_8_O_3_]^−^ (1.6) 407.0763 [M-H-2C_8_H_8_O_3_-H_2_O]^−^ (2.2) 289.0710 [M-H-C_8_H_8_O_3_-C_15_H_14_O_6_]^−^ (−0.7)	0–4
22	5.69	Unknown	C_27_H_34_O_16_	[M–H]^−^	613.1795	613.1774	3.4	405.1185 [M-H-C_7_H_12_O_7_ ]^−^ (1.5) 243.0665 [M-H-C_7_H_12_O_7_-Glu]^−^ (−0.8)	0–5
23	5.78	isomer Proanhocyanidin B	C_30_H_26_O_12_	[M–H] ^−^	577.1346	577.1351	−0.8	425.0882 [M-H-C_8_H_8_O_3_]^−^ (2.1) 407.0756 [M-H-C_8_H_8_O_3_-H_2_O]^−^ (−2.7) 289.0721 [M-H-C_15_H_14_O_6_]^−^ (3.1)	0–2
24	5.80	Isomer Procyanidin B-3-*O*-gallate	C_37_H_30_O_16_	[M–H] ^−^	729.1456	729.1461	−6.9	577.1355 [M-H-C_8_H_8_O_3_]^−^ (−0.7) 407.0784 [M-H-2C_8_H_8_O_3_-H_2_O]^−^ (−2.9)	0–3
25	5.86	Cinnamyl-galloyl-hexose	C_22_H_22_O_11_	[M–H] ^−^	461.1083	461.1089	−1.3	415.1033 [M-CH_2_O_2_]^−^ (0.5) 253.0509 [M-CH_2_O_2_-Glu]^−^ (−1.2)	4–9
26	5.90	*cis*-THSG	C_20_H_22_O_9_	[M–H] ^−^ [M + H] ^+^	405.1190 407.1332	405.1191 407.1337	−0.2 1.2	243.0665 [M-H-Glu]^−^ (3.3) 225.0550 [M-H-Glu-H_2_O]^−^ (−2.9) 197.0599 [M-H-Glu-H_2_O-CO]^−^ (179)	0–9
27	5.90	Polygoacetophenoside	C_14_H_18_O_10_	[M–H] ^−^	345.0825	345.0827	−0.6	183.0293 [M-H-Glu]^−^ (0)	0–3
28	5.90	Acetyl-emodin-*O*-hexose	C_23_H_22_O_11_	[M–H] ^−^	473.1071	473.1089	−3.8	405.1182 [M-H-C_3_HO_2_]^−^ (1.5) 243.0661 [M-H-C_9_H_10_O_7_]^−^ (0.8)	0–9
29	6.00	Methoxyl-acetyl-methyljuglone-*O*-hexose	C_20_H_22_O_10_	[M–H] ^−^	421.1139	421.114	−0.2	259.0597 [M-H-Glu]^−^ (5.8)	1–9
30	6.04	isomer Tetahydroxystilbene-*O*-(malonyl)-hexose	C_23_H_24_O_12_	[M–H] ^−^	491.1193	491.1195	−0.4	445.1124 [M-H-CO-H_2_O]^−^ (3.6) 283.0603 [M-H-CO-H_2_O –Glu]^−^ (3.2)	7–9
31	6.05	Hesperetin 7-*O*-glucoside	C_22_H_24_O_11_	[M–H] ^−^	463.1245	463.1246	−0.2	419.1362 [M-H-CO_2_]^−^ (−3.3)	8–9
32	6.12	Polygonimitin C	C_26_H_32_O_14_	[M–H] ^−^	567.1723	567.1719	0.7	243.0663 [M-H-C_12_H_21_O_10_]^−^ (−4.1)	0–1
33	6.23	Polygonflavanol A	C_35_H_34_O_15_	[M–H] ^−^	693.1816	693.1825	−1.3	541.1307 [M-H-C_8_H_8_O_3_]^−^ (−2.8) 405.1199 [M-H-C_15_H_11_O_6_]^−^ (−1.5)	0
34	6.28	Procyanidin B 2-3,3′-di-*O*-gallate	C_44_H_34_O_20_	[M–H] ^−^	881.1586	881.1571	1.7	577.0968 [M-H-2C_8_H_8_O_3_]^−^ (−6.8) 407.0787 [ M-H-3C_8_H_8_O_3_-H_2_O]^−^ (−3.7)	0
35	6.32	Tetrahydroxy-phenanthrene-*O*-hexose	C_20_H_20_O_9_	[M–H] ^−^	403.1021	403.1035	−3.5	241.0512 [M-H-Glu]^−^ (−2.5)	5–9
36	6.40	Phlorizin	C_21_H_24_O_10_	[M–H] ^−^	435.1329	435.1297	7.3	227.0717 [M-H-CO-H_2_O-Glu]^−^ (−1.32)	0–9
37	6.48	Unknown	C_26_H_28_O_12_	[M–H] ^−^	531.1498	531.1508	−1.9	405.1182 [M-H-C_6_H_7_O_3_]^−^ (2.2) 243.0680 [M-H-C_6_H_7_O_3_-Glu]^−^ (−7.0)	5–9
38	6.49	Epicatechin-3-gallate	C_22_H_18_O_10_	[M–H] ^−^	441.0838	441.0827	2.5	289.0731 [M-H-C_8_H_8_O_3_]^−^ (8.6) 169.0143 [M-H-C_15_H_12_O_5_]^−^ (3.6)	0–9
39	6.51	*trans*-THSG	C_20_H_22_O_9_	[M–H] ^−^ [M + H] ^+^	405.1194 407.1338	405.1191 407.1337	0.7 0.2	243.0668 [M-H-Glu]^−^ (4.5) 225.0554 [M-H-Glu-H_2_O]^−^ (0) 197.0601 [M-H-Glu-H_2_O-CO]^−^ (1.9)	0–9
40	6.51	Unkown	C_40_H_44_O_18_	[M–H] ^−^	811.2397	811.2455	−5.9	405.1200 [M-H-Glu-C_20_H_21_O_9_]^−^ (2.2)	0–9
41	6.54	Di-emodin-Di-hexose	C_42_H_42_O_18_	[M–H] ^−^	833.2283	833.2298	−1.8	405.1196 [M-H –C_22_H_21_O_9_]^−^ (−1.2) 243.0671 [M-H –C_22_H_21_O_9_-Glu]^−^ (−1.6)	0–8
42	6.57	Emodin-*O*-(*O*-acetyl)-glucopyranoside	C_23_H_22_O_11_	[M–H] ^−^	473.1071	473.1089	−3.8	405.1185 [M-H-C_3_HO_2_]^−^ (1.5) 243.0668 [M-H-C_9_H_10_O_7_]^−^ (−2.1)	0–9
43	6.66	Tetrahydroxystilbene-*O*-(galloyl)-glucoside	C_27_H_26_O_13_	[M–H] ^−^ [M + Na] ^+^	557.1307 581.1268	557.1301 581.1266	1.0 0.3	405.1185 [M-H-C_7_H_4_O_4_]^−^ (−0.2) 313.0565 [M-H-C_14_H_12_O_4_]^−^ (1.6) 243.0662 [M-H-C_7_H_4_O_4_-Glu]^−^ (2.1) 169.0137 [M-H-C_20_H_20_O_8_]^−^ (0)	0–9
44	6.72	Nepetin-7-*O*-glucoside	C_22_H_22_O_12_	[M–H]^−^	477.1036	477.1038	−0.4	431.0993 [M-H-CO-H_2_O]^−^ (−2.1) 269.0447 [M-H-CO-H_2_O-Glu ]^−^ (3.0)	5–9
45	6.82	2, 4-Dihydroxy-6-[(1E)-2-(4-hydroxyphenyl) ethenyl] phenyl beta-D-xylopyranoside	C_19_H_20_O_8_	[M–H]^−^	375.1092	375.1085	1.9	243.0667 [M-H-C_5_H_7_O_4_]^−^ (−1.7)	0
46	6.85	isomer polygonflavanol A	C_35_H_34_O_15_	[M–H]^−^	693.1829	693.1825	0.6	567.1480 [M-H-C_6_H_6_O_3_] (−2.8) 405.1049 [M-H-C_6_H_6_O_3_-Glu] (−2.8) 125.0248 [M-H-C_29_H_28_O_12_]^−^ (−3.2)	0–7
47	7.02	isomer Tetrahydroxystilbene-*O*-(galloyl)-glucoside	C_27_H_26_O_13_	[M–H]^−^	557.1287	557.1301	−2.5	313.0557 [M-H-C_14_H_12_O_4_]^−^ (2.6) 243.0663 [M-H-C_7_H_4_O_4_-Glu ]^−^ (0) 169.0137 [M-H-C_20_H_20_O_8_]^−^ (3.0)	0–8
48	7.09	Tetrahydroxystilbene-*O*-(acetyl)-hexose	C_22_H_24_O_10_	[M–H] ^−^	447.1257	447.1297	−8.9	405.1168 [M-H-C_2_H_2_O]^−^ (−4.4) 243.0656 [M-H-C_2_H_2_O-Glu]^−^ (−0.4)	0–1
49	7.19	Dihydroquercetin	C_15_H_12_O_7_	[M–H] ^−^	303.0506	303.0510	−1.3	151.0394 [M-H-C_7_H_4_O_4_]^−^ (4.6)	3–9
50	7.28	Mludanpioside E	C_24_H_30_O_13_	[M–H] ^−^	525.1608	525.1614	−1.1	405.1212 [M-H-C_2_H_7_O_4_]^−^ (−6.1)	0
51	7.29	2,3,5,4′-tetrahydroxystilbene-2,3-*O*- β-D-glucopyranoside	C_27_H_24_O_13_	[M–H] ^−^	555.1142	555.1144	−0.4	393.0623 [M-H-Glu]^−^ (−1.8) 274.0140 [M-H-C_14_H_17_O_6_-Glu]^−^ (−7.7)	1–9
52	7.53	isomer Tetrahydroxystilbene-*O*-(galloyl)-glucoside	C_27_H_26_O_13_	[M–H] ^−^	557.1290	557.1301	−2.0	243.0675 [M-H-C_7_H_4_O_4_-Glu]^−^ (−4.9)	0–9
53	7.61	isomer Tetrahydroxystilbene-*O*-(acetyl)-hexose	C_22_H_24_O_10_	[M–H] ^−^	447.1310	447.1297	2.9	243.0675 [M-H-Glu-C_2_H_2_O]^−^ (−4.9)	1–9
54	7.64	isomer Polygonumoside E	C_19_H_22_O_9_	[M–H] ^−^	393.1188	393.1191	−0.8	231.0674 [M-H-Glu]^−^ (−3.9)	0–1
55	7.68	Emodin-1-*O*-glucoside	C_21_H_20_O_10_	[M–H] ^−^	431.0986	431.0984	1.8	269.0453 [M-H-Glu]^−^ (1.1) 240.0440 [M-H-Glu-CHO]^−^ (7.1)	0–9
56	8.00	Di-THSG	C_40_H_42_O_18_	[M–H] ^−^	809.2307	809.2298	1.1	647.1782 [M-H-Glu]^−^ (−1.9) 485.1278 [M-H-2Glu]^−^ (−7.4) 405.1214 [M-H-C_20_H_20_O_9_]^−^ (4.2)	0–3
57	8.13	isomer Di-THSG	C_40_H_42_O_18_	[M–H] ^−^	809.2296	809.2298	−0.2	647.1758 [M-H-Glu]^−^ (1.6) 485.1245 [M-H-2Glu]^−^ (−0.6) 405.1216 [M-H-C_20_H_20_O_9_]^−^ (4.3)	0–9
58	8.15	Emodin-8-*O*-β-D-hexose-sulfate	C_21_H_20_O_13_S	[M–H] ^−^	511.0549	511.0552	−0.6	431.1000 [M-H-SO_3_]^−^ (−3.7) 269.0445 [M-H-SO_3_-Glu]^−^ (−1.9)	0–1
59	8.15	5,7-dihydroxyflavone	C_15_H_10_O_4_	[M–H] ^−^	253.0502	253.0506	−1.6	224.0461 [M-H-CHO]^−^ (−8.0)	6–9
60	8.20	N-trans-feruloyl tyramine	C_18_H_19_NO_4_	[M–H] ^−^	312.1238	312.1241	−1.0	297.1012 [M-H-CH_3_]^−^ (1.7) 190.0517 [M-H-CH_3_-C_8_H_7_O]^−^ (−0.6)	0–9
61	8.22	Tetrahydroxystilbene-2-*O*-(coumaroyl)-glucoside	C_29_H_28_O_11_	[M–H] ^−^	551.1555	551.1559	−0.7	405.1159 [M-H-C_9_H_6_O_2_]^−^ (−6.7) 243.0670 [M-H-C_9_H_6_O_2_-Glu]^−^ (5.3)	0–4
62	8.34	Tetrahydroxystilbene-2-*O*-(feruloyl)-hexose	C_30_H_30_O_12_	[M–H] ^−^	581.1661	581.1664	−0.5	405.1206 [M-H-C_10_H_8_O_3_]^−^ (4.9) 243.0665 [M-H-C_10_H_8_O_3_-Glu]^−^ (3.3)	0–6
63	8.40	trans-N-Feruloyl-3-*O*-methyldopamine	C_19_H_21_NO_5_	[M–H] ^−^	343.1362	343.1347	4.4	327.1111 [M-H-CH_3_]^−^ (0.3)	0
64	8.89	Torachrysone-8-*O*-D-glucoside	C_20_H_24_O_9_	[M–H] ^−^ [M + Na] ^+^	407.1344 431.1309	407.1348 431.1313	−1.0 −0.9	245.0822 [M-H-Glu]^−^ (3.3) 230.0578 [M-H-Glu-CH_3_]^−^ (−0.4)	0–9
65	8.91	Cirsimarin	C_23_H_24_O_11_	[M–H] ^−^	475.1235	475.1246	−2.4	245.0823 [M-H-C_9_H_10_O_7_]^−^ (−1.6)	0–3
66	8.99	Emodin-8-*O*-β-D-glucoside	C_21_H_20_O_10_	[M–H] ^−^ [M + Na] ^+^	431.0985 455.0946	431.0984 455.0949	0.2 −0.7	269.0458 [M-H-Glu]^−^ (3.0) 225.0558 [M-H-Glu-CO_2_]^−^ (2.7)	0–9
67	9.40	Emodin-8-*O*-(6′-*O*-malonyl)-β-D-glucoside	C_24_H_22_O_13_	[M–H] ^−^ [M + Na] ^+^	517.0986 541.0956	517.0988 541.0953	−0.4 0.6	473.1095 [M-H-CO_2_]^−^ (2.3) 269.0462 [M-H-C_3_H_2_O_3_-Glu]^−^ (4.5)	0–2
68	9.53	Torachrysone-8-*O*-(6 -acetyl)-glucopyranoside	C_22_H_26_O_10_	[M–H] ^−^	449.1451	449.1453	−0.4	245.0821 [M-H-C_2_H_3_O-Glu]^−^ (2.9) 230.0583 [M-H-C_2_H_3_O-Glu-CH_3_]^−^ (1.7)	0
69	9.77	2-Hydroxyemodin	C_15_H_10_O_6_	[M–H] ^−^	285.0394	285.0405	−3.9	241.0525 [M-H-CO_2_]^−^ (7.9)	0–9
70	9.89	Physcion-8-*O*-β-D-glucoside	C_22_H_22_O_10_	[M–H] ^−^ [M + Na] ^+^	445.1135 469.1112	445.1140 469.1105	−1.1 1.5	283.0622 [M-H-Glu]^−^ (5.7) 240.0435 [M-H-Glu-CO-CH_3_]^−^ (5.0)	0–9
71	9.89	Tetrahydroxystilbene-*O*-(malonyl)-hexose	C_23_H_24_O_12_	[M–H]^−^	491.1195	491.1195	0	283.0605 [M-H-C_9_H_12_O_7_]^−^ (−0.4)	0–9
72	9.89	Questin	C_16_H_12_O_5_	[M–H] ^−^ [M + H] ^+^	283.0600 285.0770	283.0612 285.0757	−4.2 4.6	240.0421 [M-H-CH_3_-CO]^−^ (−3.7)	0–9
73	10.11	Emodin-*O*-(*O*-acetyl)-glucopyranoside	C_23_H_22_O_11_	[M–H] ^−^	473.1103	473.1089	3.0	269.0457 [M-H-C_2_H_3_O-Glu]^−^ (−0.7)	1–9
74	10.21	Nootkatone	C_15_H_22_O	[M + H] ^+^	219.1735	219.1743	−3.7		0–9
75	10.48	Unknown	C_18_H_34_O_5_	[M–H] ^−^	329.2337	329.2333	1.2	211.1331 [M-H-C_6_H_14_O_2_]^−^ (−1.4)	0–9
76	10.50	Physcion-8-*O*-(6′-*O*-acetyl)-β-D-glucoside	C_24_H_24_O_11_	[M–H] ^−^	487.1243	487.1246	−0.6	283.0599 [M-H-C_2_H_3_O-Glu]^−^ (2.9) 240.0435 [M-H-C_2_H_3_O-Glu-COCH_3_]^−^ (5.0)	0
77	10.50	isomer questin	C_16_H_12_O_5_	[M–H] ^−^	283.0613	283.0612	0.4	240.0428 [M-H-CH_3_-CO]^−^ (2.1)	0–1
78	10.58	Torachrysone-*O*-acetyl-glucopyranoside	C_22_H_26_O_10_	[M–H] ^−^	449.1471	449.1453	1.0	245.0838 [M-H-C_2_H_3_O-Glu]^−^ (−7.7)	1–5
79	11.03	Unknown	C_16_H_18_O_5_	[M–H] ^−^	289.1064	289.1081	−5.9	221.1191 [M-H-C_3_HO_2_]^−^ (−3.6)	0–9
80	12.23	Unknown	C_26_H_30_N_2_O	[M + H] ^+^	387.2460	387.2431	7.5		0–9
81	12.65	Unknown	C_18_H_31_N_5_O_5_	[M + H] ^+^	398.2403	398.2398	−1.3		0–8
82	12.79	isomer acety-aloe-emodin	C_17_H_12_O_6_	[M–H] ^−^	311.0555	311.0561	−1.9	240.0420 [M-H-CH_3_CO-CO]^−^ (3.3)	4–9
83	13.44	Emodin	C_15_H_10_O_5_	[M–H] ^−^	269.0456	269.0455	0.4	241.0507 [M-H-CO]^−^ (2.5) 225.0554 [M-H-CO-O]^−^ (0.9)	0–9
84	14.87	Unknown	C_16_H_37_N_3_O_2_	[M + H] ^+^	304.2985	304.2959	−8.5		0–9
85	16.39	Polygonumate	C_16_H_22_O_4_	[M + Na] ^+^	301.1398	301.1410	−4.0		0–9
86	17.18	Unknown	C_17_H_12_O_6_	[M–H] ^−^	311.2024	311.2017	2.2	149.0952 [M-H-Glu]^−^ (−9.4)	0–9
87	19.78	Unknown	C_38_H_42_O_10_	[M + H] ^+^	659.2845	659.2851	−0.9		0–9

**Figure 2:**
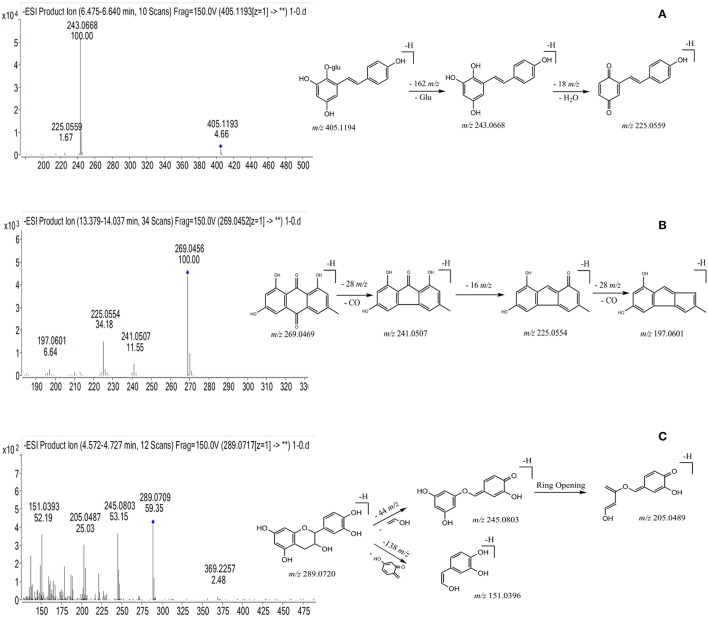
The chemical structure, typical mass spectra and proposed fragmentation pathways of three types of chemical components from PMR. **(A)** THSG; **(B)** emodin; **(C)** catechin.

Peak 39 produced precursor ions at *m/z* 405.1194 [M-H]^−^ (C_20_H_22_O_9_) and *m/z* 407.1138 [M+H]^+^, and in the negative MS/MS spectrum, fragmentation of this molecule generated product ions at *m/z* 243.0668 (C_14_H_11_O_4_) by losing a glucoside (162 Da), at *m/z* 225.0554 (C_14_H_9_O_3_) by losing a H_2_O (18 Da), and at *m/z* 197.0601 (C_13_H_9_O_2_) by losing a CO (28 Da), respectively. Peak 39 was confirmed as *trans-*THSG by comparing with the mass data of reference standard. The proposed fragmentation pathway is shown in Figure 2A. Peak 26 showed precursor ions of *m/z* 405.1190 ([M-H]^−^) and 407.1332 ([M+H]^+^), respectively, and the fragment ions at *m/z* 243.0665 (C_14_H_11_O_4_), 225.0552 (C_14_H_9_O_3_), 197.0599 (C_13_H_9_O_2_) were found. The *m/z* values of peak 26 were similar to those of peak 39, thus it was deduced as *cis*-THSG, an isomer of *trans*-THSG. Stilbene derivatives were also detected. For example, peaks 56 and 57 were established as C_40_H_42_O_18_, as they gave [M-H]^−^ ion at *m/z* 809.2307 and 809.2296, respectively. The fragment ions of peak 56 at *m/z* 647.1782, *m/z* 485.1278 and peak 57 at *m/z* 647.1758, *m/z* 485.1245 indicated successive loss of two glucosides (162 Da). Product ions at *m/z* 405.1214 and *m/z* 405.1216 were also found for peaks 56 and 57, respectively. All the results suggest that peaks 56 and 57 correspond to *di*-THSG and an isomer of *di*-THSG, respectively.

The molecular formula of peak 83 was established as C_15_H_10_O_5_, based on the detected precursor ion at *m/z* 269.0456 ([M-H]^−^) in negative mode. Fragment ions at *m/z* 241.0507 and *m/z* 225.0554 were deduced to be generated by successive loss of a –CO (28 Da) and a –O (16 Da). Based on these data as compared with the reference standard, peak 83 was confirmed as emodin (Figure 2B). Compound 66 produced a precursor ion at *m/z* 431.0985 ([M-H]^−^) (C_21_H_20_O_10_), and the fragmentation of this molecule produced product ions at *m/z* 269.0458 resulting from the loss of a glucoside (162 Da), at *m/z* 225.0558 resulting from a further loss of a –CO (28 Da). After comparing with the MS/MS spectra of emodin and emodin-8-*O*-β-D-glucoside, compound 66 was identified as emodine-8-*O*-β-D-glucoside. Compound 55 produced precursor ions [M-H]^−^ at *m/z* 4310.986 (C_21_H_20_O_10_), and diagnostic ions at *m/z* 269.0453, while *m/z* 240.0440 corresponded to the loss of a glucoside and a –CHO (29 Da). Taken together, this data indicates compound 55 should be a isomer of emodine-8-*O*-β-D-glucoside, and it was therefore identified as emodin-1-*O*-glucoside.

Compound 13 was found to have a retention time of 4.61 min, producing molecular ions of *m/z* at 289.0720 ([M-H]^−^) and 291.0861 ([M+H]^+^) (C_15_H_14_O_6_). It produced fragment ions at *m/z* 245.0803, 205.0489 and 151.0396 in the MS/MS spectrum (Figure 2C). Based on this data and previous literature reports, it could be deduced as catechin (Figure 2C). Peaks 9 and 16 gave [M-H]^−^ ions at *m/z* at 577.1353 (C_30_H_26_O_12_) and 577.1349, respectively. In the MS/MS spectrum, peak 9 gave dominant ions at *m/z* 425.0863 with a loss of C_8_H_8_O_3_ (152 Da), at *m/z* 407.0773 with a loss of H_2_O (18 Da), and *m/z* at 289.0726 (C_15_H_14_O_6_) was observed; Peak 16 produced dominant ions at *m/z* 425.0871, 407.0776 and 289.0735, implying a disintegration similar to the compound represented by peak 9. After comparing with the reference standard, peak 9 was confirmed to be proanthocyanidin B1, and compound 16 was identified as proanthocyanidin B2.

#### Multivariate statistical analysis

**Figure 3:**
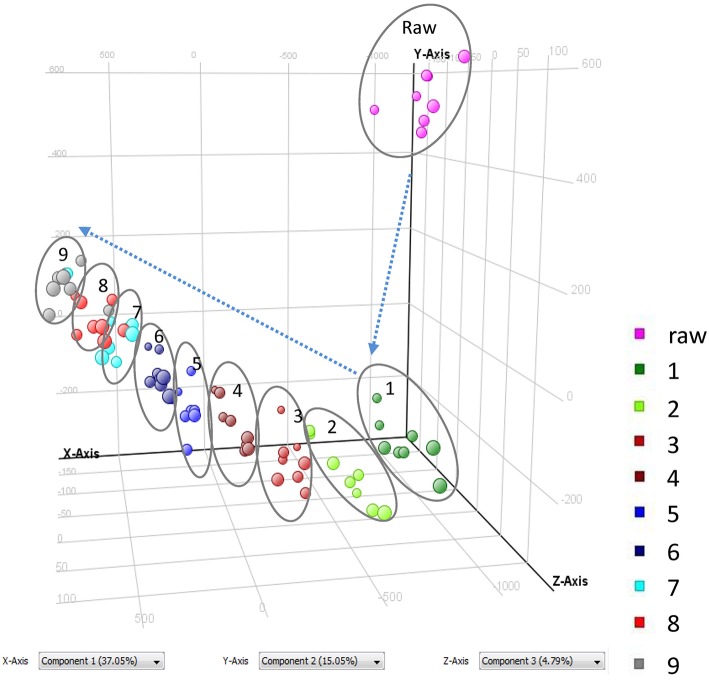
PCA score plots of RPMR and PPMR samples based on untargeted metabolomics analysis.

As shown in Figure 1 and Table 1, nine cycles processing qualitatively and progressively changed the chemical profile of PMR. A total of 68 chemical components were detected in RPMR. However, they were gradually depleted by the processing. For example, 61 and 36 of them were detected in PPMR1 and PPMR9, respectively. Meanwhile, 19 chemicals were newly detected after the processing. In order to further visualize the differences in overall chemical profiles between RPMR and PPMR, Principal Component Analysis (PCA) and Volcano Plot were used to process the MS data of untargeted metabolomics. PCA score plots clearly show PPMR plots distinct from RPMR plots; in other words, processing categorically changed the chemical profile of PMR. Furthermore, the plots from PPMR1 to PPMR9 gradually shift away from RPMR plots (Figure 3). This tendency demonstrates that the number of cycles has a critical influence on the chemical components in the final product: more changes in the chemical profile occurred with more processing cycles.

**Figure 4:**
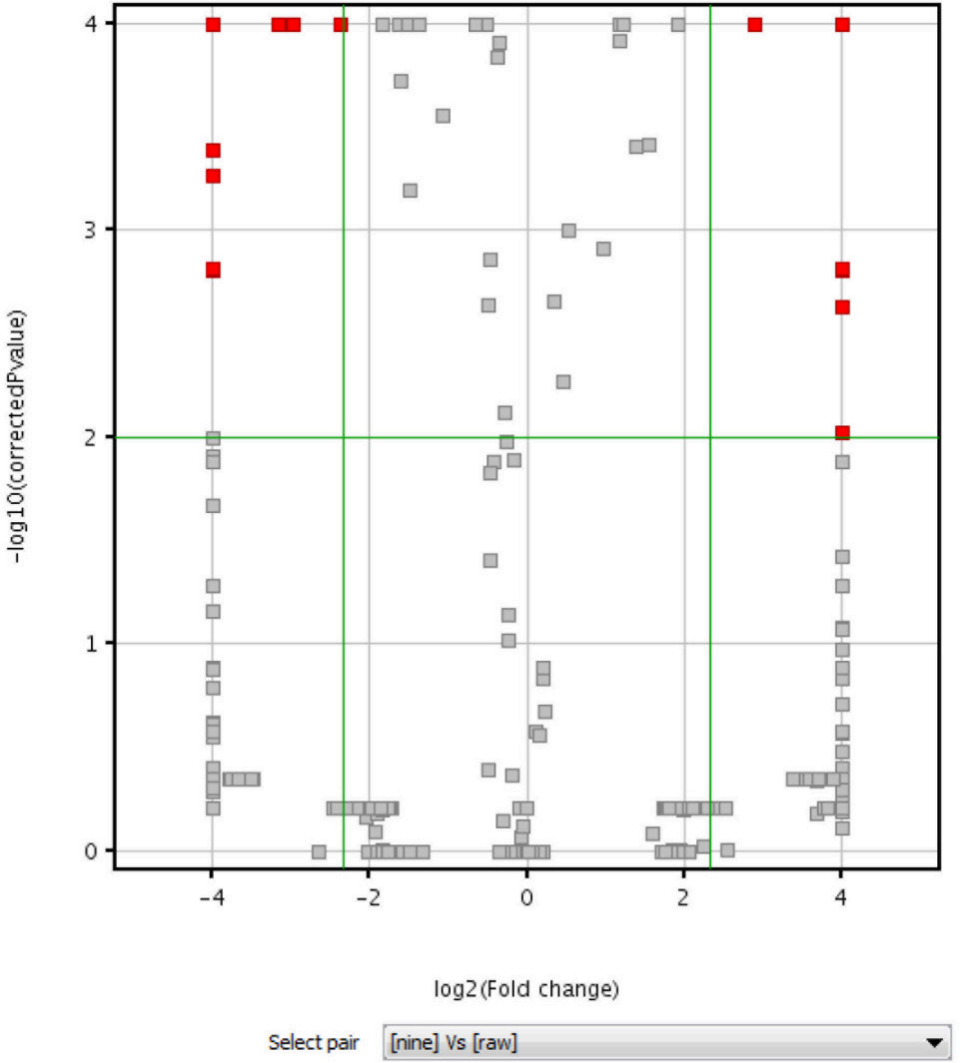
Volcano plot of raw (RPMR) and processed PMR (PPMR9) samples based on untargeted metabolomics analysis.

Volcano plot analysis was then employed to explore the chemical markers that contributed most to the difference in chemical profiles between RPMR and PPMR. Each point on the volcano plot was based on both *p*-value and fold-change values, and in this study these two values were set at 0.05 and 3.0, respectively. The points which satisfy the condition *p* < 0.05 and fold change > 3.0 appear in red and are marker candidates, whereas the others appear in gray. The software displayed the results of the markers in red as a *p*-value table with molecular weights, retention times, *p*-values and fold changes, and the contributing components were thus showed by this *p*-value table. Here, RPMR and PPMR9 were selected for the filtering analysis. Finally, 18 compounds, namely cephulac (1), gallic acid (4), 3-hydroxybenzoic acid (8), catechin (13), liquiritigenin-hexose-xyl/ara (17), isomer polygonimitin C (19), cinnamyl-galloyl-hexose (25), methoxyl-acetyl-methyljuglone-*O*-hexose (29), isomer tetahydroxystilbene-*O*-(malonyl)-hexose (30), emodin-*O*-(*O*-acetyl)-glucopyranoside (42), tetrahydroxystilbene-*O*-(galloyl)-glucoside (43), nepetin-7-*O*-glucoside (44), isomer tetrahydroxystilbene-*O*-(galloyl)-glucoside (47), 2,3,5,4′-tetrahydroxystilbene-2,3-O-beta-D-glucopyranoside (51), isomer tetrahydroxystilbene-*O*-(acetyl)-hexose (53), 5,7-dihydroxyflavone (59), emodin-*O*-(*O*-acetyl)-glucopyranoside (73) and isomer acety-aloe-emodin (82) were explored as the chemical markers most responsible for the differences between RPMR and PPMR9 (Figure 4).

### Targeted metabolomics analysis

#### UHPLC-QqQ- MS/MS conditions

To further explore how processing cycles quantitatively affect the chemical components of PMR, 12 chemicals, namely *trans*-THSG, *cis*-THSG, emodin, physcion, emodin-8-*O*-β-D-glucosides and physcion-8-*O*-β-D-glucosides, gallic acid, proanthocyanidin B1, proanthocyanidin B2, epicatechin, catechin and epicatechin-gallate, were selected as the analytes for targeted metabolomics analysis. These chemicals were not only detected as major chemical components but also reported to be the major bioactive chemical components in PMR (Yao et al., 2006; Han et al., 2013; Lin et al., 2015b). Furthermore, the UPLC-QTOF-MS/MS analysis preliminarily indicated that their contents were substantially changed by the processing.

**Figure 5:**
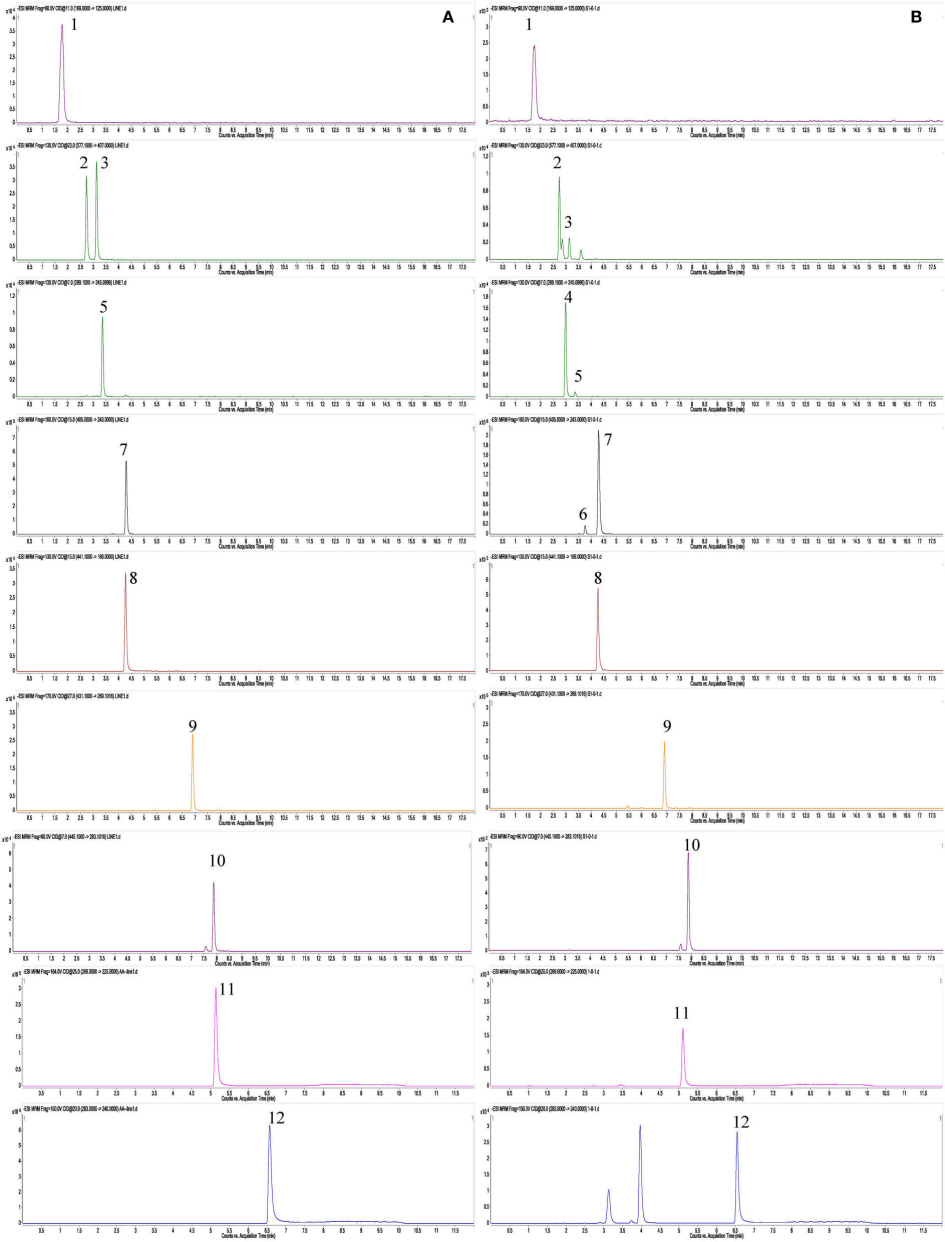
MS spectrums of 12 targeted metabolomics with MRM mode. **(A)** Reference standard; **(B)** sample (RPMR). 1, gallic acid; 2, proanthocyanidin B1; 3, proanthocyanidin B2; 4, catechin; 5, epicatechin; 6, *cis*-THSG; 7, *trans*-THSG; 8, epicatechin gallate; 9, emodin-8-*O*-β-D-glucoside; 10, physcion-8-*O*-β- D-glucoside; 11, Emodin; 12, Physcion.

In this study, UPLC-QqQ-MS/MS was used for quantitative analysis of the targeted metabolomics. Multiple reaction monitoring (MRM) is a powerful quantitative mode of UPLC-QqQ-MS/MS, for which both quadrupoles are programmed at selective scanning, allowing only one ion pair (precursor and product ions) to be detected. As two stages of mass selectivity are utilized, little interference from the background matrix exists, resulting in high specificity and sensitivity (Liang et al., 2013; Li et al., 2014). On our previous study on PMR by UHPLC-QqQ-MS/MS, chromatography conditions (mobile phase, gradient program, flow rate, injection volume), and mass parameters (ion mode, dry gas flowrate and temperature, sheath gas flowrate and temperature, nebulizer pressure, capillary voltage, dwell time) were investigated, and finally two different conditions were optimized to achieve satisfactory separation for the 12 chemicals due to their different chemical properties (Liang et al., 2018). In detail, the first set of conditions was suitable for 10 chemical components, namely gallic acid (4), proanthocyanidin B1 (9), proanthocyanidin B2 (16), *cis*-THSG (26), *trans*-THSG (39), catechin (13), epicatechin (20), epicatechin-3-gallate (38), emodin-8-*O*-β-D-glucoside (66), and physcion-8-*O*-β-D-glucoside (70). A second set of analytical conditions was developed for emodin (83) and physcion because they could not be readily ionized under the first set of conditions. The MRM fragments and Collision voltage (eV) of each analyte were individually optimized by Mass Hunter Optimizer Software (Aglient Technologies, Inc. 2010, Version B.03.01) (Table 2). For example, the ion [M-H]^−^ (*m/z* 125.0) of gallic acid was observed to be specific, stable and abundant. Thus, the ion of *m/z* 125.0 was selected as the precursor ion of gallic acid. The ion pairs of the other 11 chemicals were also similarly optimized. The MS spectrums of the analytes are shown in Figure 5.

**Table 2:** MRM conditions in UPLC-QqQ-MS/MS analysis and method validation for quantitative determination.

**Analyte**	**MRM**	**Collision voltage (eV)**	**Calibration curve**			**Sensitivity (ng/mL)**		**(RSD, %)**						
								**Repeat-ability (*n* = 6)**	**Precision (*****n*** = **6)**		**Spike Recovery (n** = **3)**			**Stability (72 h, *n* = 7)**
			**Range (ng/mL)**	**Equation**	***R*^2^**	**LOD**	**LOQ**		**Intra-day**	**Inter-day**	**Low**	**Middle**	**High**	
Gallic acid	169.0→125.0	11	80–4000	*y* = 79.28x−14.86	0.9968	17.94	56.96	2.84	2.17	5.79	106.88 (0.87)	104.22 (3.85)	112.40 (2.18)	7.95
Proanthocyanidin B1	577.1→407.0	23	80–2,000	*y* = 68.49x−1084.17	0.9959	12.50	15.66	4.96	1.13	3.56	113.48 (7.66)	105.21 (0.42)	102.97 (5.04)	6.07
Proanthocyanidin B2	577.1→407.0	23	80–2,000	*y* = 78.28x−1088.89	0.9979	8.43	11.01	7.42	4.29	2.52	99.74 (7.16)	111.41 (8.21)	100.49 (11.86)	9.44
Epicatechin	289.1→245.1	7	40–1,000	*y* = 38.44x−203.28	0.9916	4.08	38.96	6.25	5.59	11.74	89.75 (11.98)	106.90 (3.54)	96.42 (6.38)	7.30
Epcatechini-3-gallate	441.1→169.0	15	20–400	*y* = 125.61x−300.88	0.9916	4.81	7.61	2.00	2.79	3.56	100.23 (1.02)	93.55 (1.39)	93.78 (1.46)	3.09
*trans*-THSG	405.0→243.0	15	160–8,000	*y* = 288.41x−11468.51	0.9951	2.14	3.37	1.49	1.29	3.48	96.23 (5.62)	98.23 (7.38)	93.92 (4.80)	5.90
Emodin-8-*O*-β-D-glucoside	431.1→269.1	27	40–2,000	*y* = 556.32x−6573.43	0.9943	1.88	2.30	1.05	1.09	11.42	88.98 (4.00)	94.85 (4.66)	94.90 (6.70)	5.40
Physcion-8-*O*-β-D-glucoside	445.1→283.1	7	40–2,000	*y* = 89.55x−643.74	0.9953	4.57	6.85	2.59	3.09	8.03	93.32 (8.90)	98.60 (8.00)	92.25 (2.49)	10.34
Emodin	269.0→225.0	25	40–2,000	*y* = 758.58x−4801.43	0.9992	2.53	3.28	0.98	1.06	1.07	108.12 (1.23)	105.40 (1.58)	107.03 (0.58)	5.43
Physcion	283.0→240.0	20	40–2,000	*y* = 200.82x−410.28	0.9965	1.69	5.18	1.55	2.74	1.01	109.14 (3.06)	108.77 (2.00)	106.25 (1.58)	6.09

#### Quantitative method validation

The linearity, sensitivity, precision, repeatability, stability and recovery of quantitative method validation are summarized in Table 2. Due to the lack of reference standards, *cis*-THSG and catechin were semi-quantified referred to their isomers, *trans*-THSG and epicatechin, as shown in Table 2. The results showed a good liner relationship over the concentration range of each analyte, with correlation coefficients of determinations R^2^ all >0.9900. The LODs of all analytes were <17.94 ng/mL, while the LOQs were <56.96 ng/mL. The overall RSDs of intra-day and inter-day variations were not more than 5.59% and 11.74%, respectively. The spike recovery RSDs ranged from 88.98 to 113.48%, which were acceptably accurate. Stability criteria were satisfied as the RSDs were no more than 10.34% in 72 h. All these results showed that the established methods were suitable to analyze all the 12 chemical compounds; details are shown in Table 2.

#### Quantitative results

**Figure 6:**
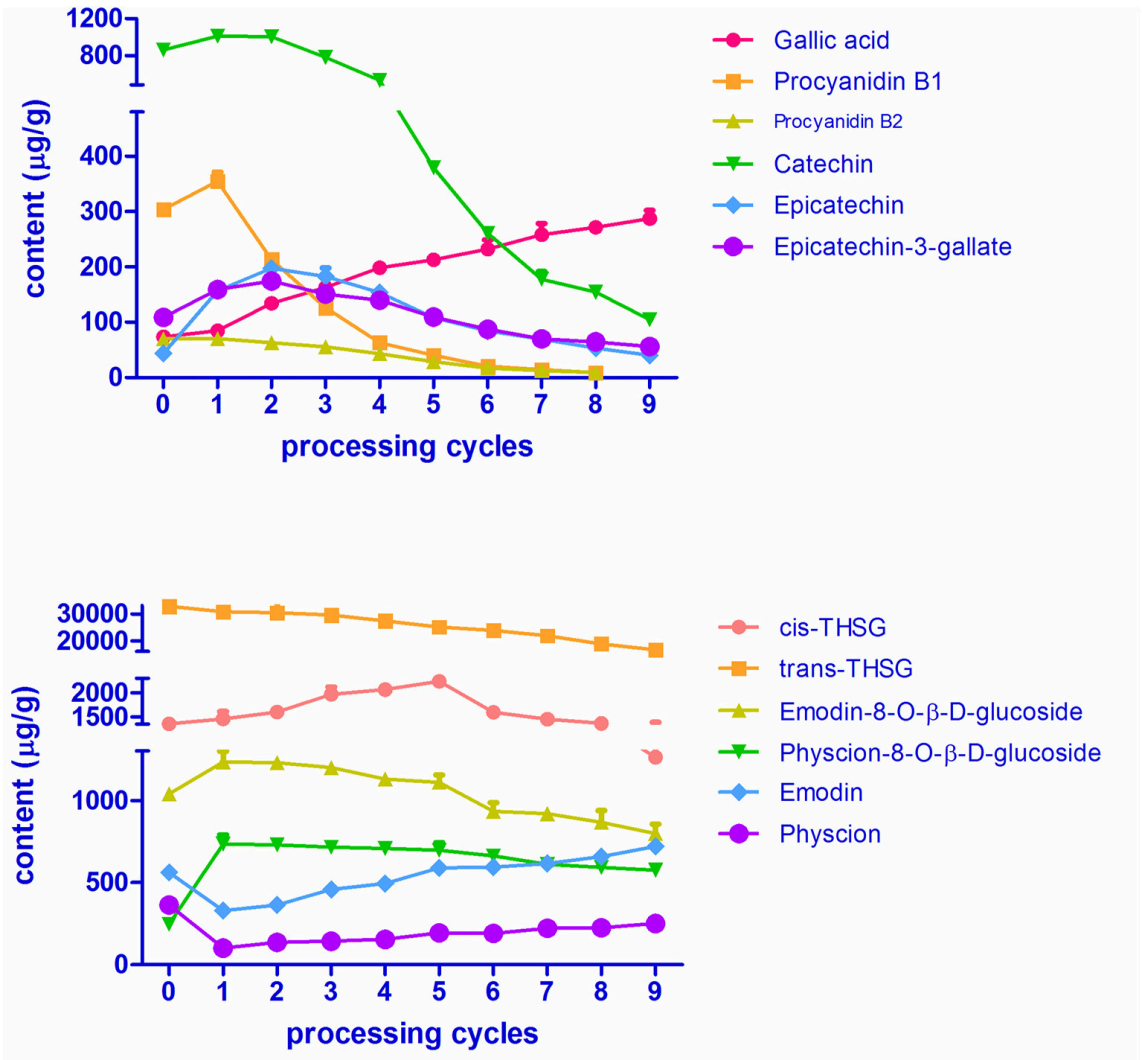
Contents of the 12 targeted secondary metabolites in RPMR and PPMR.

The variations in contents of the 12 chemicals over RPMR and PPMR are shown in Figure 6 and Table S1. Data in the table is an average of nine replicates (three samples of each processing cycle, each sample was analyzed for three times). The RSD values of nine replicates were within 9.85%, which indicate that both of the variations at each processing cycle and variations of the same samples were acceptable. The contents of two stilbene glucosides, namely *trans-*THSG and *cis-*THSG, changed significantly after RPMR was processed. The content of *cis*-THSG was 1252.28 ± 45.70 μg/g in RPMR. It first increased gradually, peaked in PPMR5 at 2066.31 ± 57.88 μg/g, and then decreased step by step to 1195.41 ± 111.92 μg/g after the ninth processing cycle; this final content was, lower than that of RPMR. In contrast, *trans-*THSG decreased consistently and regularly after each processing cycle, from 32675.01 ± 1102.04 μg/g in RPMR to 16661.65 ± 769.66 μg/g in PPMR9.

The contents of two combined anthraquinones, namely emodin-8-*O*-β-D-glucosides and physcion-8-*O*-β-D-glucosides, increased after the first cycle,from 1039.48 ± 2.23 μg/g and 228.51 ± 10.84 μg/g in RPMR to 1265.23 ± 69.56 μg/g and 734.55 ± 55.28 μg/g in PPMR1, respectively. With further processing, their contents decreased to a final content of 799.70 ± 55.32 μg/g and 574.21 ± 27.95 μg/g in PPMR9, respectively, after the ninth cycle. Free anthraquinones, including emodin and physcion, presented an opposite tendency, decreased after the first cycle and then increasing with further processing cycles. For example, the contents of emodin were 562.47 ± 8.95 μg/g, 328.81 ± 14.06 μg/g, and 720.81 ± 8.55 μg/g in the sequence of RPMR, PPMR1, and PPMR9 samples, respectively, while the contents of physcion were 363.44 ± 9.87 μg/g, 101.47 ± 6.07 μg/g, and 251.04 ± 6.75 μg/g in the same sequence of RPMR and PPMR samples (Table S1).

In the case of the six polyphenols, five of them (proanthocyanidin B1, proanthocyanidin B2, catechin, epicatechin, and epicatechin-3-gallate) increased after the first processing cycle compared to raw PMR. The content of the first three compounds started to decrease in the second cycle, whereas epicatechin and epicatechin-gallate still increased in the second cycle and began to decrease in the third cycle. In details, the content of catechin increased from 862.65 ± 43.07 μg/g in RPMR to 1010.02 ± 12.68 μg/g after being processed for one cycle, then decreased gradually after each cycle, and decreased to 104.42 ± 4.04 μg/g after nine cycles. While the variation in the contents of proanthocyanidin B1 and proanthocyanidin B2 are of the same tendency, and their contents first increased to 354.75 ± 17.10 μg/g and 70.81 ± 1.70 μg/g in PPMR1, respectively; and then decreased with the increasing of processing cycles, and finally cannot be detected after nine cycles processing. The contents of epicatechin and epcatechini-3-gallate continuously increased in the first two processing cycles, reaching its highest level of 197.43 ± 9.39 μg/g and 174.17 ± 10.63 μg/g, respectively; after that the content decreased after each cycle, finally reaching a content of 40.30 ± 1.02 μg/g and 56.12 ± 3.24 μg/g, after the last cycle, respectively. The other water-soluble constituent gallic acid increased gradually after each processing cycle, from 73.53 ± 2.67 μg/g in RPMR to 287.07 ± 14.72 μg/g in PPMR9 samples (Table S1).

In summary, the contents of gallic acid continuously increased, while *trans*-THSG continuously decreased, after each processing cycle. For emodin and physcion, the contents were first decreased and then increased with the increasing of processing cycles. In terms of the other eight analytes, namely *cis*-THSG, emodin-8-*O*-β-D-glucosides and physcion-8-*O*-β-D-glucosides, catechin, epicatechin, epicatechin-3-gallate, proanthocyanidin B1 and proanthocyanidin B2, the contents were first increased and then decreased after the increase of processing cycles. The above quantitative results not only show the changing tendency of the contents of these bioactive compounds with processing cycles, but also are helpful for further deduction of transformation mechanisms involved in the processing chemistry.

### Chemical transformation mechanisms

**Figure 7:**
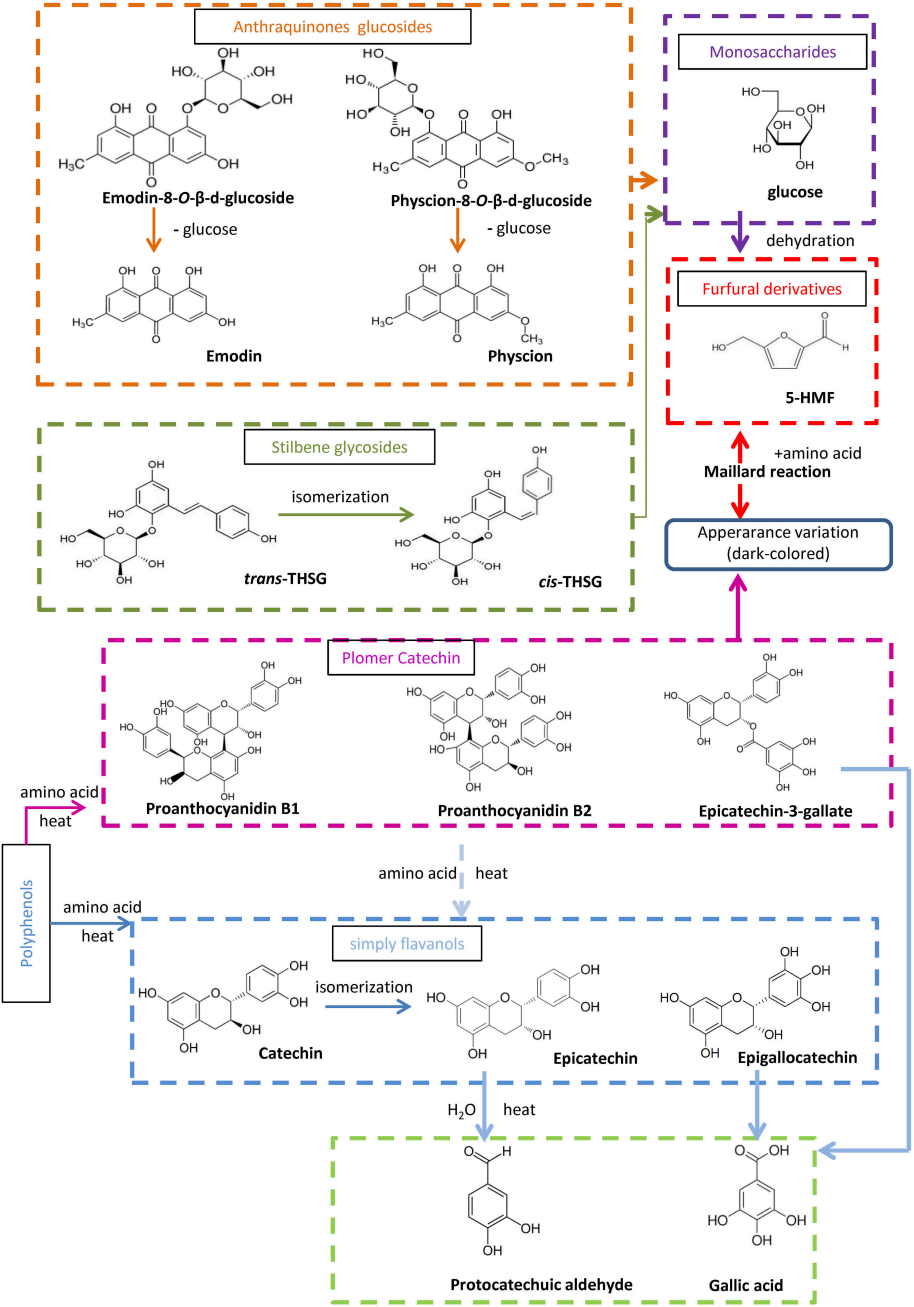
Proposed processing-induced chemical transformation mechanisms of secondary metabolites in PMR. Solid arrows, prone to happen; dotted arrows, speculated/less likely to happen.

The targeted and untargeted metabolomics results provide abundant information to discuss potential mechanisms involved in the processing chemistry of PMR (Figure 7). With regard to the two stilbene glucosides, three reactions could be involved in the variation observed. First, *trans-*THSG is easily isomerized to *cis*-THSG when exposed to heat and light as in the processing (Likhtenshtein, 2009), such that further processing would result in an increase of *cis-*THSG in the first five processing cycles. Second, both *cis-*THSG and *trans-*THSG are readily hydrolyzed, such that the steaming would decrease their content. In addition, the occurrence of 5-HMF suggest that the Maillard reaction was involved in the processing of PPMR (Liu et al., 2009).

According to the untargeted metabolomics results, emodin-8-*O*-(6′-*O*-malonyl)-β-D-glucoside, emodin-*O*-(*O*-acetyl)-glucoside, and physcion-8-*O*-(6′-*O*-acetyl)-β-D-glucoside occurred in RPMR, but were undetected after the first two processing cycles. We deduced that they were hydrolyzed to emodin-8-*O*-β-D-glucosides and physcion-8-*O*-β-D-glucosides, as both the latter were quickly increased after the first processing cycle. They would then be further hydrolyzed to emodin or physcion by the processing (Wianowska, 2014). As a result, free anthraquinones increased after each processing cycle.

It has been reported that condensed tannins can undergo acid-catalyzed cleavage in the presence of (or in the presence of an excess of) a nucleophile (Nonaka et al., 1982; Torres et al., 2002). In other words, condensed polymers are depolymerized to oligomer and monomers under thermal and acidic conditions. In the case of PPMR, all six water soluble components increased after the first processing cycle. In addition, the oligomers and monomers, except for gallic acid, were also unstable in thermal and acidic conditions, so they were subject to structural transformation after further processing, which caused catechin to be transformed to its isomeric compound epicatechin (Ross et al., 2011). Besides the isomerization, polymer and monmers were further depolymerized, so that the monomer protocatechuic aldehyde could, finally, be found after processing, and gallic acid, also a final derived monomer, accumulated after each processing cycle.

Some studies have suggested that THSG is a toxic component of PMR (Wu et al., 2012; Meng et al., 2017), in which case, less THSG is safer for human consumption. Our results showed that the content of *trans*-THSG gradually decreased after each processing cycle and was lowest after nine cycles, while the content of *cis*-THSG initially increased and only began decreasing after the fifth processing cycle, reaching its lowest content after nine cycles. These results indicate that from the perspective of toxicity of PMR, ideally nine cycles, and certainly more than five cycles, is necessary to produce a safer PPMR for clinic use. However, this still warrants further confirmation by toxicodynamic studies. In addition, as mentioned above, RPMR and PPMR are used for distinct medicinal purposes, while RPMR is used to resolve toxin and free the stool, PPMR is regarded as a traditional tonic for its rejuvenation effects. As combined anthraquinones function as a purgative and are, thus, not suitable as a dietary supplement (Zhao and Xiao, 2010), we deduced that lower combined anthraquinones contents is more appropriate for seeking the supplement effect of PPMR. The content of combined anthraquinones was consistently decreased by the processing, and was lowest in PPMR9. These results indicate that nine cycles of processing might be necessary for its clinic function since combined anthraquinones presented their lowest contents in the last processing cycle. Besides the above mentioned contents variation, results show that the contents of other bioactive compounds in this study, such as catechin, gallic acid, and proanthocyanidin B2, changed after being processed, which may also linked with the changing of therapeutic effect (Yao et al., 2006; Han et al., 2013; Lin et al., 2015b). However, this still needs furtherly verified by pharmacodynamics studies. In summary, based on processing chemistry of PMR, nine cycles processing is necessary to produce a safer, more effective form of PPMR for clinical and home use, whereas further pharmacodynamics and toxicodynamic studies should be carry out to verify it.

## Conclusion

In this study, targeted and untargeted metabolomics analyses were integrated to investigate the processing chemistry of PMR. The results demonstrate that the processing by nine cycles of steaming and drying qualitatively and quantitatively alters the chemical profiles of PMR. Several mechanisms, namely hydrolysis, dehydration, isomerization and the Maillard reaction, were potentially involved in the chemical variation. The qualitative and quantitative data further suggest that the nine cycles of processing might be necessary for the preparation of PPMR, and the processing producers cannot be abbreviated to modern one processing cycle, as PPMR that has been processed nine times shows significant differences in its chemical profile and less potentially disruptive chemicals. The research results indicate that the metabolomics strategy could comprehensively characterized the processing chemistry of herbal medicines, thereby contributing to understand the scientific basis of herbal medicine processing.

## Author contributions

LL and JX conceived and designed the experiments. LL and W-WZ performed the experiment. LL analyzed the data. H-BC and Z-ZZ guided the experiment. LL wrote the paper. JX and EB revised the paper. Z-ZZ acquired funding for the research. All authors read and approved the final manuscript.

### Conflict of interest statement

The authors declare that the research was conducted in the absence of any commercial or financial relationships that could be construed as a potential conflict of interest.

## Supplementary material

Figure S1 The appearance of RPMR and PPMR1-9.

Figure S2 Typical UPLC-QTOF-MS/MS chromatograms of secondary metabolites in PMR with different mobile phases. **(A,D)** RPMR; **(B,E)** PPMR1; **(C,F)** PPMR9; **(A–C)** 0.1% formic acid–water and 0.1% formic acid–acetonitrile; **(D–F)** 3 mM ammonium acetate-water and methanol.

Table S1 Quantitative results of analysis of secondary metabolites in RPMR and PPMR (μg∙g-1) (*n* = 9). Data in the table is an average ± SD of nine replicates; ^*^ represent undetected.
