# Supplementary figures and images for: Integrating Targeted and Untargeted Metabolomics to Investigate the Processing Chemistry of Polygoni Multiflori Radix
Source: Front Pharmacol. 2018 Aug 28;9:934. doi: 10.3389/fphar.2018.00934 (PMC6121093; doi:10.3389/fphar.2018.00934)

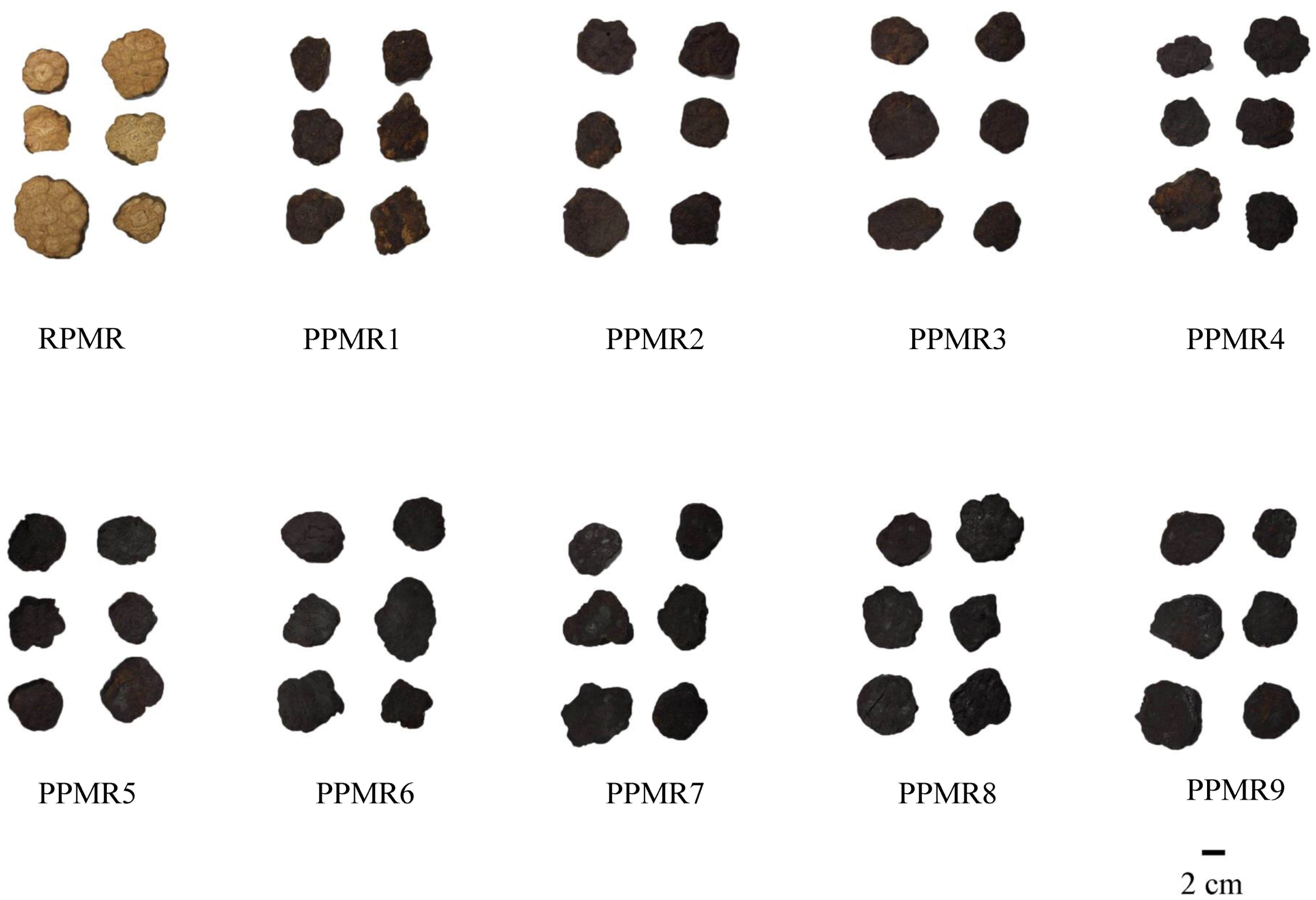

Supplement: Figure S1 — The appearance of RPMR and PPMR1-9. [file Image_1.TIF]

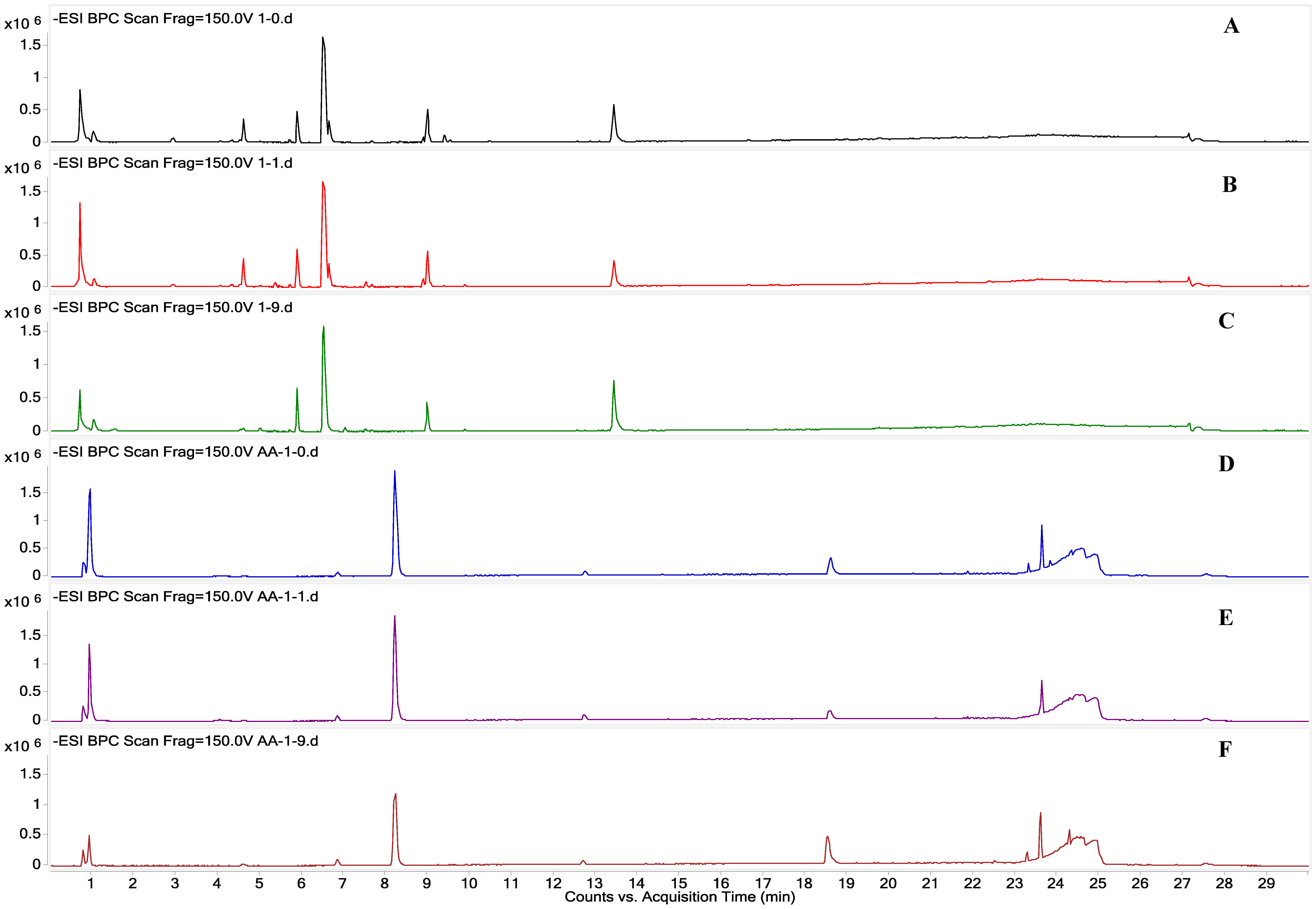

Supplement: Figure S2 — Typical UPLC-QTOF-MS/MS chromatograms of secondary metabolites in PMR with different mobile phases. (A,D) RPMR; (B,E) PPMR1; (C,F) PPMR9; (A–C) 0.1% formic acid–water and 0.1% formic acid–acetonitrile; (D–F) 3 mM ammonium acetate-water and methanol. [file Image_2.TIFF]
