# Supplementary material for: Integrating Targeted and Untargeted Metabolomics to Investigate the Processing Chemistry of Polygoni Multiflori Radix
Source: Front Pharmacol. 2018 Aug 28;9:934. doi: 10.3389/fphar.2018.00934 (PMC6121093; doi:10.3389/fphar.2018.00934)
Supplement: Table S1 — Quantitative results of analysis of secondary metabolites in RPMR and PPMR (μg∙g-1) (n = 9). Data in the table is an average ± SD of nine replicates; * represent undetected. [file Table_1.DOCX]

**Table S1. Quantitative results of analysis of secondary metabolites in RPMR and PPMR (μg·g^-1^) (n=9)**

| **Secondary metabolites** | **Processing cycles** | | | | | | | | | |
| --- | --- | --- | --- | --- | --- | --- | --- | --- | --- | --- |
|  | **RPMR** | **PPMR-1** | **PPMR-2** | **PPMR-3** | **PPMR-4** | **PPMR-5** | **PPMR-6** | **PPMR-7** | **PPMR-8** | **PPMR-9** |
| Gallic acid | 73.53±2.67 | 84.47±5.29 | 134.46±3.83 | 163.23±2.25 | 198.61±6.47 | 212.92±4.88 | 231.98±16.85 | 258.18±20.31 | 271.77±11.26 | 287.07±14.72 |
| Proanthocyanidin B1 | 304.03±5.93 | 354.75±17.10 | 213.09±10.07 | 126.14±7.44 | 63.05±1.56 | 40.52±1.64 | 20.50±0.71 | 14.18±0.97 | 9.08±0.15 | * |
| Proanthocyanidin B2 | 70.23±4.24 | 70.81±1.70 | 63.18±3.68 | 55.23±5.44 | 42.93±3.65 | 28.98±0.75 | 17.10±0.98 | 12.74±0.63 | 9.62±0.44 | * |
| Catechin | 862.65±43.07 | 1010.02±12.68 | 1003.04±13.28 | 780.65±16.29 | 528.89±18.39 | 379.18±7.92 | 260.82±11.50 | 177.47±14.06 | 154.39±1.31 | 104.42±4.04 |
| Epicatechin | 43.37±1.16 | 157.27±3.08 | 197.43±9.39 | 183.05±15.01 | 153.94±3.86 | 108.52±7.38 | 84.32±4.59 | 69.24±4.55 | 53.09±1.38 | 40.30±1.02 |
| Epcatechini-3-gallate | 109.03±6.04 | 159.38±4.59 | 174.17±10.63 | 150.91±5.98 | 139.94±9.86 | 109.28±8.22 | 87.04±2.65 | 70.24±4.74 | 64.83±4.01 | 56.12±3.24 |
| *cis*-THSG | 1252.28±45.70 | 1469.65±136.53 | 1600.44±141.82 | 1966.26±159.37 | 2066.31±57.88 | 2241.49±89.17 | 1596.00±74.66 | 1453.99±108.09 | 1365.56±125.39 | 1195.41±111.92 |
| *trans*-THSG | 32675.01±1102.04 | 30744.70±2048.98 | 30429.92±2969.25 | 29550.48±387.13 | 27368.83±556.01 | 25053.72±1258.73 | 23852.30±887.88 | 21855.66±944.23 | 18778.02±822.15 | 16661.65±769.66 |
| Emodin-8-*O*-β-​D-​glucoside | 1039.48±2.23 | 1265.23±69.56 | 1231.02±20.78 | 1199.16±8.50 | 1130.09±26.00 | 1109.99±46.76 | 933.32±53.92 | 919.80±6.64 | 865.72±73.59 | 799.70±55.32 |
| Physcion-8-*O*-β-​D-​glucoside | 228.51±10.84 | 734.55±55.28 | 730.52±29.15 | 714.93±11.93 | 708.51±9.64 | 697.83±46.13 | 661.46±42.21 | 612.23±32.49 | 592.57±11.10 | 574.21±27.95 |
| Emodin | 562.47±8.95 | 328.81±14.06 | 362.96±16.82 | 459.08±11.42 | 494.92±18.73 | 589.54±27.02 | 594.98±16.13 | 617.24±26.41 | 659.06±9.61 | 720.81±8.55 |
| Physcion | 363.44±9.87 | 101.47±6.07 | 135.43±10.18 | 142.80±11.52 | 153.42±5.70 | 193.61±5.04 | 191.22±7.99 | 220.56±7.97 | 225.86±2.79 | 251.04±6.75 |

Data in the table is an average ± SD of nine replicates; * represent undetected.
